# The outcome of applications for restoration to the Medical Register following disciplinary erasure

**DOI:** 10.1177/0025817220960605

**Published:** 2020-12-14

**Authors:** Bryony KP Milroy, Timothy J David, Sarah Ellson

**Affiliations:** 1 Manchester University NHS Foundation Trust, Manchester, UK; 2 Faculty of Biology, Medicine and Health, University of Manchester, Manchester, UK; 3 Fieldfisher LLP, Manchester, UK

**Keywords:** General Medical Council, Medical Practitioners Tribunal Service, erasure, restoration, fitness to practise

## Abstract

In the period 1 January 2012 to 30 June 2020, 76 doctors whose names/entries had been erased from the UK Medical Register by a disciplinary tribunal applied for restoration, and 23 out of 76 (30.3%) applications granted. In 5 of the 53 of those refused restoration, the tribunal suspended indefinitely the right to make further applications. The most frequent reasons for refusal were failure to demonstrate insight (seen in 96%), failure to demonstrate remediation (seen in 79%), and failure to demonstrate that knowledge and skills were up to date (24.5%). Success was more common in UK graduate applications (14/29 – 48.3%) than non-UK graduate applications (9/37 – 24.3%), and in those legally represented (16/29 – 55.2%) than in those without legal representation (7/29 – 24.1%), but the data does not indicate the reasons for these differences. Disciplinary erasure need not necessarily be for life as doctors who learn from their experience, change their ways, and provide evidence of genuine insight and remediation along with up to date knowledge and skills can successfully be reinstated on the register.

## Introduction

In order to practise medicine in the UK, the doctor’s name must be included in the Medical Register held by the General Medical Council (GMC). Disciplinary action by the GMC can result in the removal of a doctor’s name (referred to as erasure) from the Register, which halts the doctor’s ability to practise medicine in the UK.

Erasure is not necessarily the end of the road. Doctors who have been erased by a disciplinary tribunal of the GMC or by the Medical Practitioners Tribunal Service (MPTS), which has managed doctors’ disciplinary tribunals since 2012, can apply for their names to be restored once five years have elapsed.

Erasures that follow an MPTS tribunal are referred to as “disciplinary erasures”, as distinct from administrative or voluntary erasures, which are not the subject of the present study. There are brief published case reports of individual examples of restoration following disciplinary erasure,^1^,^2^ and in a historical review of restoration there is mention that between 1858 and 1990 some 42% (247 out of 595) of doctors whose names had been erased for disciplinary reasons were restored.^3^ However, there has been no published study and accordingly we have studied the outcome of such applications, considered by an MPTS tribunal from January 2012 to June 2020.

## Regulatory framework

The backdrop to restoration applications is the three-pronged statutory overarching objective as set out in s 1 Medical Act 1983 to protect, promote, and maintain the health, safety, and well-being of the public; to promote and maintain public confidence in the medical profession; and to promote and maintain proper professional standards and conduct for members of that profession. The central question for the tribunal is whether or not the doctor concerned is fit to practise without restrictions.^4^,^5^ It is for the applicant seeking restoration to demonstrate that he or she is fit to practise. If there is a decision to restore registration, that registration would be unrestricted, so, for example, a decision to restore registration could not be accompanied by the application of conditions to the registration.

The MPTS in its guidance for tribunals considering restoration applications has advised that there will be cases where restoration is generally unlikely to be in line with the overarching objective, such as murder, rape or sexual assault by penetration, sexual offences involving children, or adults with a mental disorder impeding choice, offences involving human trafficking, slavery, servitude and forced or compulsory labour, and extortion and blackmail. This would be irrespective of the length of time that has elapsed and whether or not there is strong evidence that the doctor has demonstrated insight and maintained their clinical knowledge and skills. Caution is also advised where a doctor has a suspended sentence which remains in force, or where a doctor’s name is on the Sex Offenders Register, because it would be inappropriate to hold unrestricted registration in these circumstances and restoration cannot be granted subject to conditions.

Any tribunal decision regarding a restoration application is likely to give careful consideration to all the circumstances of the case, including, but not limited to the following:

- the circumstances which led to the erasure;
- the reasons given by the earlier panel for the decision to direct erasure;
- whether or not the doctor has any insight into the matters that led to the erasure;
- what the doctor has done since their name was erased from the Medical Register; and
- steps taken by the doctor to keep their medical knowledge and skills up to date.

When considering whether to restore a doctor to the register the tribunal cannot go behind the original tribunal’s findings on facts, impairment, and sanction but it will first consider the evidence of insight, remorse, and remediation against the backdrop of the matters which led to the erasure, and makes findings about those matters. Then, if positive findings are made, the tribunal steps back and balances those findings against each of the three limbs of the overarching objective, and considers the case overall, including the length of time which has elapsed since erasure. When deciding whether or not the doctor is fit to practise, the tribunal needs to consider whether the restoration of the doctor would promote and maintain public confidence and proper professional standards so that, notwithstanding the serious nature of the original matters which led to the erasure, the overarching objective to protect the public would be achieved.

Before making a decision about restoration, a tribunal can require assessments of the doctor’s health, English language ability, and/or professional performance.^6^,^7^

Tribunal decisions have to give clear reasons for their decisions and demonstrate that all three aspects of the overarching objective were considered. Full details of the approach taken when considering restoration have been set out by the MPTS.^8^

If an application for restoration has been unsuccessful, the doctor cannot make a further application until 12 months have elapsed from the date of the last application. Although the GMC has the power to appeal a decision to grant restoration, there is no statutory right of appeal for a doctor who is refused. However, the doctor may challenge the decision by way of judicial review. If a doctor makes two unsuccessful applications, the right to make further applications can be suspended indefinitely by the tribunal that considers the doctor’s most recent restoration application. There is a statutory right of appeal against the decision to suspend the doctor’s right to re-apply indefinitely. A doctor can apply for the suspension to be reviewed by a tribunal after three years have elapsed from the date of the decision to suspend indefinitely the right to apply. If the suspension is not lifted the doctor can apply again to have it lifted after a further three years.

The GMC can appeal decisions to permit restoration decisions, where it considers that a decision to restore a doctor is not sufficient for the protection of the public. The GMC is required to notify the Professional Standards Authority of certain decisions by an MPTS tribunal, including those to restore a doctor’s name to the register, and the Authority can refer the case to the High Court of Justice in England and Wales if they consider a restoration decision should not have been made.

In the past, restoration after disciplinary erasure was possible after only a relatively short period (as little as 10 months), causing considerable concern (which reached a peak in 1998) that the public were being insufficiently protected.^9^ However, since that time there have been important changes to the regulation of doctors in the UK. From 3 August 2000, new legislation meant that erasure was for a minimum of five years. In addition, this legal change was accompanied by cases that have established a clear test for restoration, which had never really been scrutinised in the 1990s.

## Subjects and methods

The materials used in this study were the written determinations concerning MPTS hearings to consider applications for restoration to the register held between 1 January 2012 and 30 June 2020. These were obtained in two different ways. The first method is that cases were identified by BM who searched for the words “restoration” and “restore” in all the MPTS practitioners tribunal determinations downloaded each year from the MPTS website by TD. The second method was that upon request, the MPTS supplied all the determinations they could identify concerning restoration applications in the years 2012–2019. Cases concerning applications for restoration following voluntary erasure or administrative erasure were excluded. Cases included for study were those in which a doctor applied for restoration to the register following what is described as “disciplinary erasure”. These are cases that have been referred to the MPTS by the GMC because of concerns about a doctor’s fitness to practise. Determinations of MPTS tribunals are published on the MPTS website and thus enter the public domain. Matters relating to health are redacted. A small number (67 in the years 2012–2019) of MPTS tribunal cases solely concerned health matters, and these determinations are not publicly available for study.

## Results

Of a total of 76 applications for restoration, 23 (30.3%), 19 males, 4 females, resulted in restoration being granted, and 53 (69.7%), 46 males, 7 females, were refused. In addition, there was one additional doctor who (unsuccessfully) applied for a review of the indefinite suspension of their right to re-apply for restoration.

### Number of previous applications

Regarding the 23 successful applications, in 19 this was the first application, in 2 it was a second application, in 1 it was a third application, and in 1 it was a fourth application. In four cases, before granting restoration the applicant had to undergo an assessment of their professional performance^6^,^7^ to ensure that their knowledge and skills were up to date, with a successful outcome in all four cases.

Regarding the 53 unsuccessful applications, in 37 this was the first application, in 13 it was a second application, and in 3 it was a third application.

### Duration of qualification and time from erasure to application for restoration

The duration of qualification (from qualification to application hearing) ranged from 12 to 42 years (median 23, mean 25.2, SD 8.6 years) for those granted restoration and ranged from 15 to 61 years (median 28, mean 29.4, SD 10.2 years) for those refused restoration. The time from erasure to restoration application ranged from 6 to 15 years (median 8, mean 8.4, SD 2.0 years) for those granted restoration and ranged from 5 to 40 years (median 7, mean 9.0, SD 6.0 years) for those refused restoration.

### Location of primary medical qualification (PMQ)

Regarding the 23 successful applications, 14 (60.9%) obtained their PMQ in the UK and 9 (39.1%) obtained their qualification outside the EEC.

Regarding the 53 unsuccessful applications, 17 (32.1%) obtained their PMQ from the UK, 4 (7.5%) obtained their PMQ from another EEC country, and 32 (60.4%) obtained their PMQ from outside the EEC.

### Presence and legal representation

Regarding the 23 successful applications, 22/23 (95.7%) applicants attended the hearing, and 16/23 (69.6%) were legally represented.

Regarding the 53 unsuccessful applications, 46/53 (86.8%) applicants attended the hearing, and 16/53 (30.2%) applicants were legally represented.

### The problem(s) resulting in erasure

In some cases, there was more than one problem that resulted in erasure.

Regarding the 23 successful applications, 16 (69.6%) resulted from dishonesty, 6 (26.1%) resulted from criminal behaviour, and 1 (4.3%) resulted from deficient professional performance.

Regarding the 53 unsuccessful applications, 39 (73.6%) resulted from dishonesty, 8 (15.1%) resulted from criminal behaviour, 14 (26.4%) resulted from deficient professional performance, and 11 (20.8%) resulted from sexually motivated misconduct.

### The reasons given for refusal of restoration

In most cases, there was more than one problem that resulted in refusal. In 51/53 (96.2%) cases, failure to demonstrate insight was given as a reason. In 42/53 (79.2%) cases, a failure to demonstrate remediation was given as a reason. In 13/53 (24.5%), a failure to demonstrate that their knowledge and skills were up to date was given as a reason. Additional reasons were continuing evidence of dishonesty (six cases) and inadequate English language skills (one case). The exceptionally serious nature of the allegations at the time of erasure was a cause for concern in some cases, but this was only given as a contributory reason for refusal to grant restoration in one case.

### Indefinite suspension of right to apply for restoration

In 5 of the 53 (9.4%) of those refused restoration, the tribunal decided to suspend indefinitely the right to make further applications for restoration.

In one further case, two refusals to grant restoration were accompanied by an indefinite suspension of the right to make further applications, and this was later followed by two unsuccessful applications to review the indefinite suspension.

### Erasure occurring twice

In one case, a doctor had been erased twice. Following the first erasure which resulted from a criminal conviction, the doctor was restored to the register on a second application for restoration. Later, a second erasure resulted from dishonesty, and two applications for restoration (made during the period of this study) were unsuccessful.

## Discussion

Unpublished data suggest that in the order of 636 doctors’ names were erased in the eight-year period 2012–2019, which is worth noting because it is evident from our study that most do not seek restoration and therefore it is only a very small number who do actually return to UK practice after erasure.

The results of the study confirm that erasure is not necessarily the end of the road, and 23/76 (30.3%) applications for restoration were successful. However, there are stiff challenges to be overcome. In particular, the need to be able to provide evidence that skills and knowledge relevant to practice in the UK are at a safe level can be difficult, given that erasure prevents clinical practice in the UK, although an erased doctor can sometimes be provided with an attachment as an observer. Success appears to depend upon an ability to network with UK doctors who are willing and able to offer support and supervision, or to practise medicine overseas and provide robust evidence of positive qualities. A number of successful applicants have plainly gone to immense lengths to move on and develop their medical careers.

Successful application was more common in UK graduate applications (14/29 – 48.3%) than non-UK graduate applications (9/37 – 24.3%), and in those legally represented (16/29 – 55.2%) than in those without legal representation (7/29 – 24.1%). The data does not indicate the reasons for these differences, but it seems reasonable to speculate that those who have been trained in the UK system, and who have had the benefit of legal advice, may have an advantage in complying with the requirements for a successful application. Our impression is that legal representation can be helpful, not merely because of advocacy at the tribunal hearing, but possibly more importantly, as a result of advice given 2–3 years ahead of an application regarding the kind of evidence that will be needed for a successful outcome. The apparent benefit of legal representation comes at a considerable price, for doctors who have been erased are not likely to be able to receive the free legal representation available to registered doctors who are members of a medical defence organisation.

At present, the GMC has the power to appeal against an MPTS tribunal decision to grant restoration. In one such case, the GMC appealed this decision, but the High Court dismissed the GMC’s appeal.^10^ The GMC subsequently appealed against the High Court’s decision. The Court of Appeal found that the MPTS tribunal had made “an error of principle” in that it had failed to properly have regard to the statutory overarching objective.^11^ In a subsequent addendum to the Court of Appeal’s decision, the matter was referred back to the original tribunal for reconsideration in the light of the Court of Appeal judgment.^12^ The final outcome was that the tribunal confirmed its original restoration decision having had regard to the overarching objective.

The unifying feature of unsuccessful outcomes is a lack of insight, commonly a serious problem that was evident at the time of erasure, often accompanied by a misguided motivation for a restoration application (i.e. doctors who still want to deny the facts and earlier findings). A quotation often referred to in restoration determinations is “Insight is most material to ensure that the doctor has realised that he has indeed gone wrong and therefore will not do anything similar in the future”.^13^ It is self-evident that if one cannot recognise that what one has done is wrong, then self-correction becomes impossible. The Sanctions Guidance for MPTS tribunals^14^ advises that a doctor is likely to lack insight if they:

- refuse to apologise or accept their mistakes;
- promise to remediate, but fail to take appropriate steps, or only do so when prompted immediately before or during the hearing;
- do not demonstrate the timely development of insight; or
- fail to tell the truth during the hearing.

It should be noted that the Sanctions Guidance cautions that “The tribunal should be aware that cultural differences and the doctor’s circumstances (e.g. their ill health) could affect how they express insight”.

Another factor common to most unsuccessful applications is a failure to demonstrate remediation. This of course requires a recognition of the errors and failings that led to the erasure outcome. The Sanctions Guidance refers to the importance of remediation thus:

Remediation is where a doctor addresses concerns about their knowledge, skills, conduct or behaviour. Remediation can take a number of forms, including coaching, mentoring, training, and rehabilitation (this list is not exhaustive),and, where fully successful, will make impairment unlikely. However, there are some cases where a doctor’s failings are irremediable. This is because they are so serious or persistent that, despite steps subsequently taken, action is needed to maintain public confidence. This might include where a doctor knew, or ought to have known, they were causing harm to patients, and should have taken steps earlier to prevent this.

Remediation needs to be relevant, measurable, and effective. Whilst the idea that some extreme behaviours may be irremediable could induce a feeling of hopelessness, particularly in cases of dishonesty where remediation is often said to be difficult to achieve, a notable finding of the present study is that of the 23 successful applications for restoration, 16 (69.6%) resulted from dishonesty. Sufficient evidence of remediation was provided in all 16. Remediation may also be hard to demonstrate in cases of sexual misconduct, but this was nevertheless achieved in all four of the successful applications which involved sexual misconduct.

Dr Paula Case, an academic lawyer, has argued that the demonstration of insight and remediation may be no more than a sham in the “redemption model” of fitness to practise, referring to a “contrived exchange of remorse, insight and remediation”.^15^ The implication is that the doctor seeking restoration needs to provide evidence that their apparent insight and remediation are genuine changes that have been achieved.

A limitation of this study is that the published determinations do not provide the age of the doctor. The determinations did not suggest that the age of the doctor was a factor in the restoration decision. A possible proxy for age was the number of years between obtaining the PMQ and applying for restoration. The data showed that the median period between obtaining the PMQ and the date of restoration hearing for those who were successful was 23 years, compared with a median age of 28 years for those who were unsuccessful, but it is not known to what extent these figures reflect the ages of the individuals.

## Conclusions

Disciplinary erasure need not necessarily be for life. The legislation which allows applications for restoration applications shows that government intended that restoration should be a potential even in the most serious of cases. People can learn from their mistakes and change their ways. The aim of restoration is to enable offending doctors an opportunity to resume worthwhile professional lives without presenting a further danger to members of the public. For those doctors who are capable of providing evidence of genuine insight and remediation, and who can demonstrate that their knowledge and skills are up to date, restoration may be seen as a way of enabling the rehabilitation of doctors and of giving them something for which to strive during the period of erasure.

## References

[1] Dyer C. Doctor who was struck off for misconduct is restored to the register after changing his ways. BMJ 2020; 368. 10.1136/bmj.m723 32094204

[2] Dyer C. Anaesthetist is second doctor this year to be restored to GMC register. BMJ 2020; 368. 10.1136/bmj.m1039 32165396

[3] Smith RG. *Medical discipline: the professional conduct jurisdiction of the General Medical Council, 1858–1990*. Chapter 7. Oxford: Oxford University Press, 1994, pp.198–220.

[4] Gomez D. The regulation of healthcare professionals: law, principle and process. 2nd ed. Chapter 34. London: Sweet & Maxwell, 2019, pp.1948–1958.

[5] Hamer K. Professional conduct casebook. 3rd ed. Chapter 71. Oxford: Oxford University Press, 2019, pp.987–1005.

[6] Southgate L Cox J David T, et al. The assessment of poorly performing doctors: the development of the assessment programmes for the General Medical Council’s Performance Procedures. Med Educ 2001; 35: 2–8. 11895250

[7] Southgate L Cox J David T, et al. The General Medical Council’s Performance Procedures: peer review of performance in the workplace. Med Educ 2001; 35: 9–19. 11895258 10.1046/j.1365-2923.2001.0350s1009.x

[8] Medical Practitioners Tribunal Service. Guidance for medical practitioners tribunals on restoration following disciplinary erasure 80485824.pdf (came into effect 31 October 2019).

[9] Smith R. GMC under the cosh. BMJ 1998; 316.

[10] *General Medical Council* v *Chandra* [2017] EWHC 2556 (Admin).

[11] *General Medical Council* v *Chandra* [2018] EWCA Civ 1898.

[12] *General Medical Council* v *Chandra* [2019] EWCA Civ 236.

[13] *The Queen on the Application of Dr Keith Bevan* v *The General Medical Council* [2005] EWHC 174 (Admin), 4 February 2005.

[14] General Medical Council and Medical Practitioners Tribunal Service. Sanctions guidance for members of medical practitioners tribunals and for the General Medical Council’s decision makers. London, General Medical Council, 18 November 2019.

[15] Case P. The good, the bad and the dishonest doctor: the General Medical Council and the ‘redemption model’ of fitness to practise. Legal Stud 2011; 31: 591–614.

